# A conservative treatment for eosinophilic cystitis

**DOI:** 10.1002/iju5.12533

**Published:** 2022-11-15

**Authors:** Franco Alchiede Simonato, Nicola Pavan, Mirko Pinelli, Gabriele Tulone, Rosa Giaimo, Anna Martorana, Alchiede Simonato

**Affiliations:** ^1^ University of Genoa Urology Clinic Genoa Italy; ^2^ Department of Surgical, Oncological and Stomatological Sciences University of Palermo Palermo Italy; ^3^ Department of Health Promotion, Mother and Child Care, Internal Medicine and Medical Specialties University of Palermo Palermo Italy

**Keywords:** conservative therapy, eosinophilic cystitis, hematuria, hyperbaric therapy, hypereosinophilic syndrome

## Abstract

**Introduction:**

Eosinophilic cystitis is a rare condition which causes common symptoms and may mimic other conditions. Eosinophilic cystitis has several causes such as hypereosinophilic syndrome, inflammatory diseases, neoplasia, parasites or fungal infection, IgE‐related diseases, Drug Reaction and Eosinophilia and Systemic Symptoms (DRESS) syndrome, or Churg‐Strauss syndrome. Therefore, differential diagnosis is difficult.

**Case presentation:**

We report the case of a middle‐aged man affected by eosinophilic cystitis with persistent hematuria and other peculiar symptoms that may be brought back to hypereosinophilic crisis.

**Conclusion:**

Conservative approach is preferred, avoiding radical cystectomy rather than corticosteroid, antihistaminic and second line therapy. Hyperbaric therapy is an innovative approach for severe relapsing gross hematuria without specific literature and should be studied for further indications.

**Abbreviations & Acronyms**
bmb: bone marrow biopsy
CSS: churg‐Strauss Ssyndrome
CT: computerized tomography
EC: eosinophilic syndrome
HES: Hypereosinophilic syndrome
TUR: transurethral resection

**Keynote message**

We present a rare case of differential diagnosis in a patient with gross hematuria and/or lower urinary tract symptoms: HES manifested as EC should be considered in differential diagnosis.

## Case report

Hypereosinophilia (HE) is an uncommon condition that occurs most frequently in middle‐aged males. It has been first classified by Hardy and Anderson in 1968^1^ and is defined as the presence of >1500 eosinophils/mm^3^ in peripheral blood in two samples taken at an interval of at least two months and/or the presence of tissue eosinophilia.^2^, ^3^ HE could be associated with many different causes, such as hypereosinophilic syndrome (HES),^2^ inflammatory diseases,^2^, ^4^ neoplasia,^2^ parasites or fungal infection,^2^, ^4^ IgE‐related diseases,^2^ DRESS syndrome,^2^ or Churg‐Strauss syndrome (CSS).^5^ It could lead to the development of severe systemic or isolated damages^2^ and/or multiple organ damages,^3^ such as eosinophilic cystitis (EC), but the pathophysiology is unknown.^3^, ^6^ EC is a relatively rare inflammatory condition of the bladder^7^ and its association with HES is far rarer.^3^ Patients commonly present with urinary frequency, dysuria, hematuria, and supra‐pubic pain, which may lead to a wrong diagnosis of a urinary tract infection.^6^, ^8^ It is difficult to distinguish EC from other forms of cystitis.^6^, ^9^

## Case

**Fig. 1:**
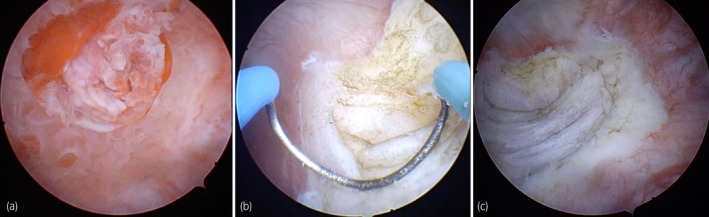
Cystoscopic images taken during a biopsy for diagnostic assessment: (a) Ulcerated pseudopolypoid lesion on the bottom of the bladder; (b) endoscopic biopsy of the postero‐lateral wall of the urinary bladder; (c) resection bed: normal tissue layers are not visible.

**Fig. 2:**
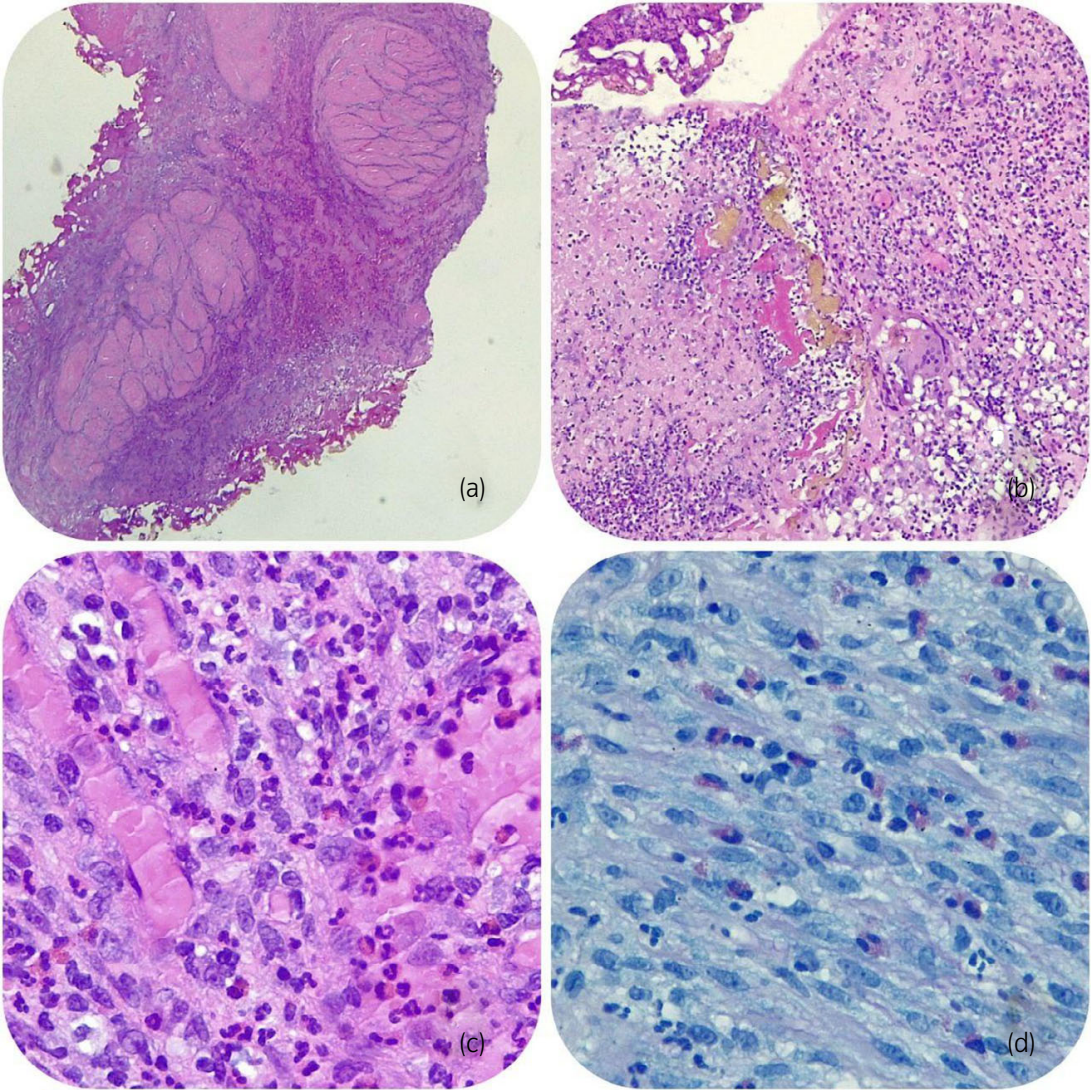
Biopsy of the posterior wall of the urinary bladder, compatible with the diagnosis of HE. (a) Severe inflammation extended to the muscular layer; (b) occasional multinucleated giant cells and foci of necrosis (hematoxylin–eosin ×100); (c) eosinophilic infiltrate (Hematoxylin–eosin ×400); (d) numerous eosinophils in chorion (Giemsa ×400).

**Fig. 3:**
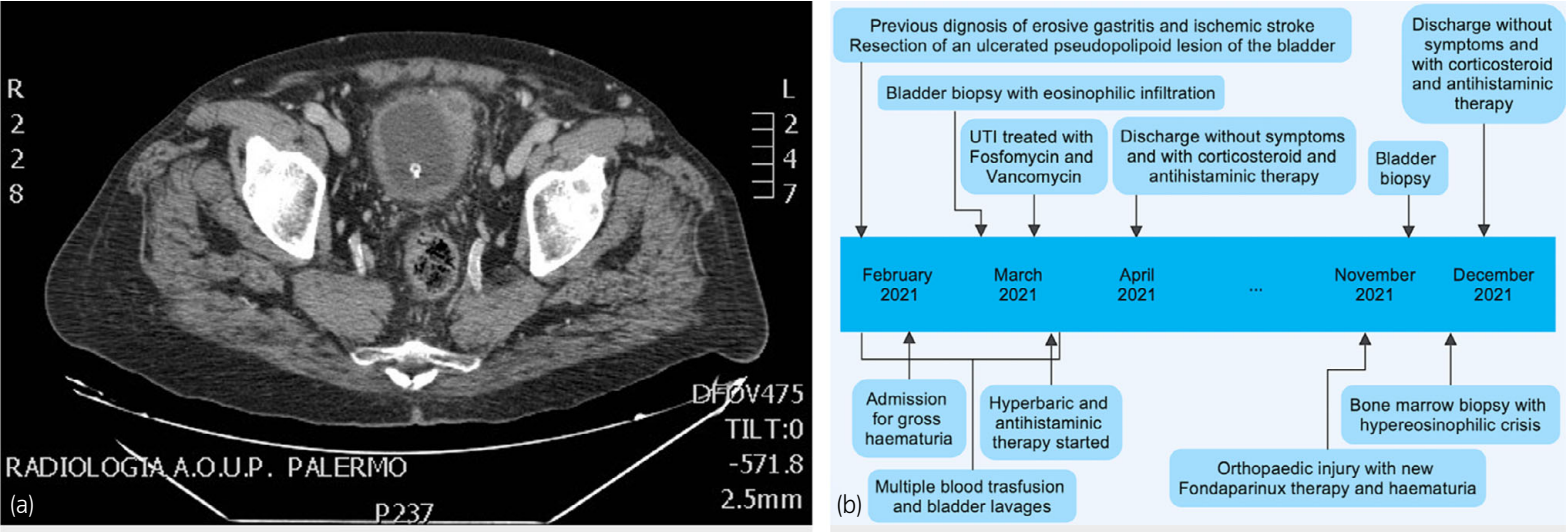
(a) Uro‐CT showing hypervascularization around the bladder, thickening of posterolateral wall, an effusion due to vesical rupture in the anterolateral wall and enlargement of locoregional lymph nodes; (b) chart diagram with time showing the clinical course with symptoms, exams, and therapies.

A 50‐year‐old patient presented with hematuria and a mass in the posterior wall of the bladder discovered with an ultrasound scan. He had already been hospitalized months earlier for an ischemic stroke treated with fondaparinux and clopidogrel for a month. Following an episode of melena, he underwent blood transfusions and performed a gastroduodenoscopy, with clinical diagnosis of erosive gastritis with a discrete eosinophilic infiltrate. The anamnesis was negative for allergies, asthma, or respiratory diseases. Physical examination revealed dark macules alongside the interdigital space of his hands. After a cystoscopy, which confirmed the ultrasound finding, he underwent an endoscopic resection of the lesion with a histological report of polypoid cystitis. Blood transfusions and hemostatic transurethral resection (TUR) surgery were performed due to incoercible hematuria and consequent anaemia. In the following, he was readmitted to the Emregency Room (ER) and underwent further blood transfusions and hemostatic TUR with new biopsy sampling. Cystoscopy showed inflammation of the mucosa and an ulcerated pseudopolypoid lesion on the bottom of the bladder (Fig. 1). The histological examination (Fig. 2) revealed a rich eosinophilic‐giant cell lympho‐granulocyte infiltrate with granulomatous aspects and some necrotic focus, in the absence of peripheral hypereosinophilia. The patient returned to the hospital for hematuria after about one week and underwent blood transfusions and hemostatic TUR with biopsy sampling again. The histological examination showed intense chronic inflammation with a large amount of eosinophilic granulocytes, in the absence of microorganisms. Uro‐computerized tomography (CT) was performed and the results showed a diffuse thickening of bladder walls, with low capacity and small lumen, enlarged regional and para‐aortic lymph nodes. Microbiological testing (Quantiferon, *Mycobacterium tuberculosis* in urine, and Schistosoma eggs in stool) were performed, all with negative results. As hematologists suggested, the study of coagulation factors and thromboelastogram (TEG) was performed in the suspicion of a hemostasis disease, but with a negative result.Due to gross hematuria, another hemostatic TUR and blood transfusions were performed. The biopsy samples were sent for microbial analysis for the search of parasites, fungi, and bacteria. The result was positivite for Klebsiella pneumoniae and *Enterococcus faecalis* on histological specimens. The infection was treated with fosfomycin (4 g/thrice a day) and vancomycin (1 g/twice a day). It was decided to avoid radical cystectomy in the young patient. Rheumatological and neurological counseling were required: the rheumatologic etiology was neglected, but antinuclear antibodies (ANA) were found with fine‐speckled pattern positivity (1:320); the neurological examination revealed polyneuropathy of upper and lower limbs. After finding total IgE 11 times higher than the cut‐off, therapy with Cetirizine (10 mg/die) was set and hyperbaric therapy (2 h/day for 5 days) was performed. Positive urine culture confirmed the infection on the histological finding; therefore, cortisone therapy was postponed. He was discharged with a bladder catheter, cetirizine (10 mg/day), and prednisone (30 mg/day) therapy, in order to keep the disease inactive. The macules and gross hematuria disappeared and polyneuropathy improved, but gross hematuria reappeared with new fondaparinux therapy for orthopedic injury. Triphasic contrast CT highlighted flogosis of the maxillary sinuses and low‐capacity urinary bladder with thickening of the posterolateral wall and an effusion nearby the resection area (Fig. 3). Hematochemical analysis showed iron‐deficiency anemia and total IgE 3 times higher than the upper limit. An urinary bladder biopsy revealed a rich eosinophilic and lympho‐monocytic infiltrate without perivascular fibrinoid necrosis. Rheumatologic counseling excluded vasculitis and hematological counseling suggested to perform a bone marrow biopsy (BMB), which revealed a hypereosinophilic crisis. In addition, research for FIP1L1‐PDGFR1α fusion gene was prescribed. Corticosteroid and antihistaminic therapy was set and hematuria resolved. If symptoms reappear, cystoscopy will be performed and second line therapy will be considered.

## Discussion

In this case, the first manifestation of EC was hematuria, which is a common symptom with multiple differential diagnosis, ranging from bladder cancer to urinary infection. Bladder cancer may mimic EC with polypoid lesions and vice versa,^4^, ^9^ like in this case. Therefore, an endoscopic resection was performed, hematuria continued. Bioptic samples showed eosinophilic infiltration without peripheral eosinophilia. Hyperbaric, antihistamine and prednisone therapy was set and symptoms diminished. Hyperbaric therapy for the reduction of bleeding in EC is not supported by any evidence in literature, but can be performed in radiation cystitis.^10^ Parasites and fungal infections were excluded with negative history for recent trips and negative cultures, but biopsy and urine culture revealed a suspected urinary catheter sovra infection by *Klebsiella pneumoniae* and *Enterococcus faecalis*, not relevant for the differential diagnosis. Food or drug allergy reaction history was negative, though IgE in serum were elevated. An incompatible antibody pattern, the rheumatologic and internal medicine counseling, the CT results, and the bioptic examination were not diagnostic for CSS. There are no established guidelines for the management of EC in HES, and there is no known cure for EC^3^ except for the surgical one.^7^

Table 1 shows five EC case reports with the respective patient characteristics, clinical presentation, treatment, outcome, and follow‐up. Four out of six patients were male and two out of six were under 25‐years‐old. Only one patient was not reported with hematuria and/or eosinophilic bladder infiltration, but he and other four patients had bladder thickening. Four patients underwent prednisone therapy with positive outcome, except for one patient who reported a diminished quality of life.^8^ Nerve sparing subtotal cystectomy and radical cystectomy with orthotopic ileal neobladder with the Vescica Ileale Padovana technique were performed in only two patients with the resolution of all the symptoms.^7^ Antihistamine therapy is reported with resolution of the symptoms^4^, ^7^ especially when associated with prednisone,^4^ but one patient reported a diminished quality of life^8^ and another one relapsed after 1 month from bladder catheter removal.^7^ Treatment with benralizumab reported with a clinical improvement both in outcome and follow‐up^8^. Nonsteroidal anti‐inflammatory drugs, hydroxyurea, IFN‐alpha, mepolizumab, imatinib and other tyrosine‐kinase inhibitors, vincristine, 6‐mercaptoputine, busulfan, chlorambucil, azathioprine, cyclosporine‐A, cytarabine, methotrexate, immunoglobulins, and alemtuzumab were reported for the EC second line treatment.^2^, ^3^, ^6^, ^8^

**Table 1:** Patient characteristics, clinical presentation, treatment, outcome, and follow‐up from other case reports

Reference	Sex	Age	Clinical presentation	Treatment	Dosage	Outcome	Follow‐up
Jang2014	M	55	Gross hematuria, leukocytosis, hypereosinophilia, bladder thickening , eosinophilic bladder infiltration	Prednisone	1 mg/kg per day for 6 weeks per os	Bladder infiltration completely resolved. Partial hemological remission	Fluctuant eosinophil count for 6 months
Özdoğan2014	M	8	Terminal hematuria, dysuria, eosinophiluria, hypereosinophilia, diffuse bladder enlargement and thickening, eosinophilic bladder infiltration	Ibuprofen	10 mg/kg in 3 doses	Hematuria completely resolved Bladder thickening decreased from 11 mm to 4 mm after 4 weeks of treatment	Still asymptomatic after one year
				Desloratadine	5 mg/day		
				Clarithromycin			
Chia2016	M	73	Hematuria, dysuria, frequency, bladder thickening, eosinophilic bladder infiltration	Prednisone		Hematuria completely resolved	Still asymptomatic after 4 weeks
Rossanese 2017	M	23	Urinary storage, urinary voiding, bilateral HUN^†^, bladder thickening	Prednisone	25 mg/day	HUN^†^ resolved after 6 weeks. Bladder thickness decreased after 6 weeks.	After one month from urinary catheter removal, severe LUTS^‡^ recurred.
				Nitrofurantoine	50 mg/day		
				Levocetirizine	5 mg/day		
				Nerve sparing subtotal cystectomy with orthotopic ileal neobladder (VIP^§^ technique)		No LUTS^‡^ after 3 years. Normal renal function after 3 years. Normal erectile and ejaculatory function after 3 years.	
	M	62	3‐year history of recurrent high‐risk non‐muscle invasive bladder cancer, large erythematous bladder thickening, right HUN^†^, eosinophilic bladder infiltration	Radical cystectomy with orthotopic ileal neobladder (VIP^§^ technique)		Still asymptomatic after 2 years. No LUTS^‡^ after 2 years. No HUN^†^ after 2 years.	
Cooke2020	F	78	Dysuria, urinary frequency (20 voidings per day and 20 voidings per night), urinary emergency, urinary incontinence, pelvic pain, hematuria, eosinophilic bladder infiltration	Oral corticosteroids		No benefits reported. Self‐reported QoL continued to diminish.	
				Antihistamines			
				Montelukast			
				Antibiotic suppressive therapy			
				Antispasmodics			
				Benralizumab	30 mg s.c. every 4 weeks	Urinary frequency reduced (from 20 voidings per night to 4–8) Urinary incontinence reduced Self‐reported QoL^¶^ and UDI‐6^††^ increased	Continued cystitis with rare eosinophils, after 9 months UDI‐6^¶^ increased from 58 to 38, after 9 months 34% improvement of self‐reported QoL^¶^, after 9 months

^†^ Hydroureteronephrosis.

^‡^ Lower urinary tract symptoms.

^§^ Vescica ileale padovana.

^¶^ Quality of life.

^††^ Urogenital distress inventory.

In case of gross hematuria and/or lower urinary tract symptoms, HES manifested as EC should be considered in differential diagnosis. BMB and cystoscopy with biopsy should be performed to confirm the suspicion. Major, but not resolving, surgery should be avoided in favor of a conservative therapy. Hyperbaric therapy could be considered to treat EC hematuria and studied for additional indications, such as hematuria.

## Ethical statement

An institutional review board approval was not required as this was a single case. The patient provided informed consent.

## Conflict of interest

The authors declare no conflicts of interest associated with this manuscript.

## Registry and the Registration No. of the study/trial

Not applicable.

## Author contributions

Franco Alchiede Simonato: Conceptualization; methodology; writing – original draft; writing – review and editing. Nicola Pavan: Conceptualization; formal analysis; writing – original draft. Mirko Pinelli: Conceptualization; supervision. Gabriele Tulone: Investigation; methodology; validation. Rosa Giaimo: Methodology; supervision; validation. Annamaria Martorana: Supervision; visualization; writing – review and editing. Alchiede Simonato: Conceptualization; investigation; supervision; writing – review and editing.

## References

[1] Hardy WR , Anderson RE . The Hypereosinophilic syndromes. Ann. Intern. Med. 1968; 68: 1220–9. 5653621 10.7326/0003-4819-68-6-1220

[2] Rolla G , Fornero M . Ipereosinofilia e sindromi ipereosinofile. Riv Immunol E Allergol Pediatr. 2014: 14–24. https://www.riaponline.it/wp‐content/uploads/2017/05/04_Rolla_Immunologia‐1.pdf

[3] Jiang P , Wang C , Jin B , Lin Y , Chen S . Eosinophilic cystitis in a patient with hypereosinophila syndrome: a case report. Exp. Ther. Med. 2014; 8: 49–51. 24944595 10.3892/etm.2014.1706 PMC4061232

[4] Özdoğan EB , Tıraş Ş , Çamlar SA *et al*. An unusual cause of terminal hematuria in a child: eosinophilic cystitis. Can. Urol. Assoc. J. 2014; 8: e867–71. 25485018 10.5489/cuaj.2173 PMC4250255

[5] Heers H , Ramaswamy A , Hofmann R . A case of Churg‐Strauss syndrome (eosinophilic granulomatosis with polyangiitis) of the urinary bladder. Urology 2017; 108: 7–10. 28477943 10.1016/j.urology.2017.04.042

[6] Chia D . Eosinophilic cystitis and haematuria: case report of a rare disease and common presentation. Int. J. Surg. Case Rep. 2016; 24: 43–5. 27179336 10.1016/j.ijscr.2016.04.055 PMC4873027

[7] Rossanese M , Palumbo V , Sioletic S , Crestani A , Giannarini G , Ficarra V . Surgical treatment of eosinophilic cystitis in adults: a report of two cases and a literature review. Urol. Int. 2019; 102: 122–4. 29554647 10.1159/000485257

[8] Cooke WD , Cooke AJT . Successful treatment of eosinophilic cystitis with benralizumab. Urol. Case Rep. 2020; 33: 101379. 33102077 10.1016/j.eucr.2020.101379 PMC7574143

[9] Fuller TW , Dangle P , Reese JN *et al*. Inflammatory Myofibroblastic tumor of the bladder masquerading as eosinophilic cystitis: case report and review of the literature. Urology 2015; 85: 921–3. 25817116 10.1016/j.urology.2015.01.005

[10] Oscarsson N , Müller B , Rosén A *et al*. Radiation‐induced cystitis treated with hyperbaric oxygen therapy (RICH‐ART): a randomised, controlled, phase 2–3 trial. Lancet Oncol. 2019; 20: 1602–14. 31537473 10.1016/S1470-2045(19)30494-2

